# SDMA as a marker and mediator in cerebrovascular disease

**DOI:** 10.1042/CS20241021

**Published:** 2024-10-11

**Authors:** Alexandra Riddell, Arun Flynn, Hugo Bergugnat, Laura B. Dowsett, Alyson A. Miller

**Affiliations:** British Heart Foundation Glasgow Cardiovascular Research Centre, School of Cardiovascular and Metabolic Sciences, University of Glasgow, Glasgow, United Kingdom

**Keywords:** cerebral ischemia, cerebrovascular disease, endothelial dysfunction, methylarginine, Symmetric dimethylarginine

## Abstract

Symmetric dimethylarginine (SDMA) is a methylated derivative of arginine, generated by all cells as a by-product of cellular metabolism and eliminated via the kidney. For many years SDMA has been considered inert and of little biological significance. However, a growing body of evidence now suggests this view is outdated and that circulating SDMA levels may, in fact, be intricately linked to endothelial dysfunction and vascular risk. In this review, we specifically examine SDMA within the context of cerebrovascular disease, with a particular focus on ischaemic stroke. We first discuss pre-clinical evidence supporting the notion that SDMA has effects on nitric oxide signalling, inflammation, oxidative stress, and HDL function. We then appraise the most recent clinical studies that explore the relationship between circulating SDMA and cerebrovascular risk factors, such as chronic kidney disease, hypertension, atrial fibrillation, and atherosclerosis, exploring whether any associations may arise due to the existence of shared risk factors. Finally, we consider the evidence that elevated circulating SDMA is linked to poor outcomes following ischaemic and haemorrhagic stroke. We draw upon pre-clinical insights into SDMA function to speculate how SDMA may not only be a marker of cerebrovascular disease but could also directly influence cerebrovascular pathology, and we highlight the pressing need for more mechanistic pre-clinical studies alongside adequately powered, longitudinal clinical studies to fully evaluate SDMA as a marker/mediator of disease.

## Introduction

As one of the leading causes of morbidity and mortality, cerebrovascular disease amounts to an enormous healthcare and economic burden globally [1]. Indeed, despite intense research efforts, no specific treatments presently exist for vascular causes of dementia [2]. Equally, the management of acute ischaemic stroke relies on timely thrombolysis and thrombectomy, which are not suitable for all patients and are only recommended within a narrow time window [3,4]. Accordingly, current strategies to reduce the burden of stroke depend heavily on risk reduction via the control of modifiable cerebrovascular risk factors; although it can be challenging to grasp the particular significance of any singular risk factor for a given patient [5]. As such, there is an unmet need for both effective therapeutics that impact disease outcomes and for novel biomarkers that support the identification and stratification of individuals most at risk of cerebrovascular disease.

The pathogenesis of cerebrovascular disease is closely associated with endothelial dysfunction. The endothelium is an important source of nitric oxide (NO), a potent vasodilator derived from L-arginine by endothelial nitric oxide synthase (eNOS). NO regulates vascular tone and resting cerebral blood flow (CBF) by regulating endothelium-dependent vasodilatation through the activation of the soluble guanyl cyclase (sGC)-cyclic GMP (cGMP)-protein kinase G (PKG) pathway [6], and further contributes to brain homeostasis through its roles in angiogenesis and neurogenesis, and its anti-inflammatory and anti-thrombotic functions [7–9]. Diminished NO bioavailability is a hallmark of endothelial dysfunction, often coinciding with oxidative stress, inflammation, and a reduction of the anti-thrombotic properties of the endothelium [10]. Ultimately, this leads to alterations in the structure and function of cerebral blood vessels, dysregulation of CBF, blood–brain barrier (BBB) dysfunction and neurological damage [11,12]. Endothelial dysfunction is a feature of all major (cerebro)vascular risk factors, including hypertension [13], atrial fibrillation (AF) [14], Type 2 diabetes mellitus [15], chronic kidney disease (CKD) [16], and atherosclerosis [17], and is considered a fundamental mechanism by which these risk factors negatively impact upon brain health. Furthermore, in acute stroke endothelial dysfunction increases the severity of pathology and exacerbates neurological deficits [7,18].

Endogenous methylarginines are a group of non-proteinogenic amino acids that have been associated with endothelial dysfunction, cerebrovascular risk and stroke outcomes [19–24]. Asymmetric dimethylarginine (ADMA) has received the most attention within the scientific community due to its function as a competitive inhibitor of NOS and the growing body of evidence supporting its positive association with obesity [25], diabetes [26], myocardial infarction [27], cerebral small vessel disease [28], and stroke [19]. Although its role as a mediator of disease is yet to be fully understood, causal involvement of ADMA in the pathogenesis of hypertension [29] and atherosclerosis has been described [30]. By contrast, little is known about its structural isomer symmetric dimethylarginine (SDMA). Whilst SDMA does not directly inhibit NOS [31], evidence supports that SDMA reduces NO bioavailability and has pro-oxidative, pro-inflammatory effects, at least *in vitro*. Moreover, clinical studies have reported a positive association between elevated SDMA levels in plasma or cerebral spinal fluid (CSF) with poor outcomes following ischaemic or haemorrhagic stroke [20–24]. In this review, we consider the evidence supporting previously overlooked biological functions of SDMA and evaluate the clinical data that looks at the association of SDMA with cerebrovascular risk factors. We review the links between SDMA and cerebrovascular disease outcomes, taking a particular focus on ischaemic stroke, where most data are available. We explore why these associations might exist and speculate on potential directions of future research which could be undertaken to provide further insight into the significance of SDMA as a marker, or perhaps even a mediator, of endothelial dysfunction and cerebrovascular pathology.

## The synthesis and metabolism of dimethylarginines

**Figure 1:**
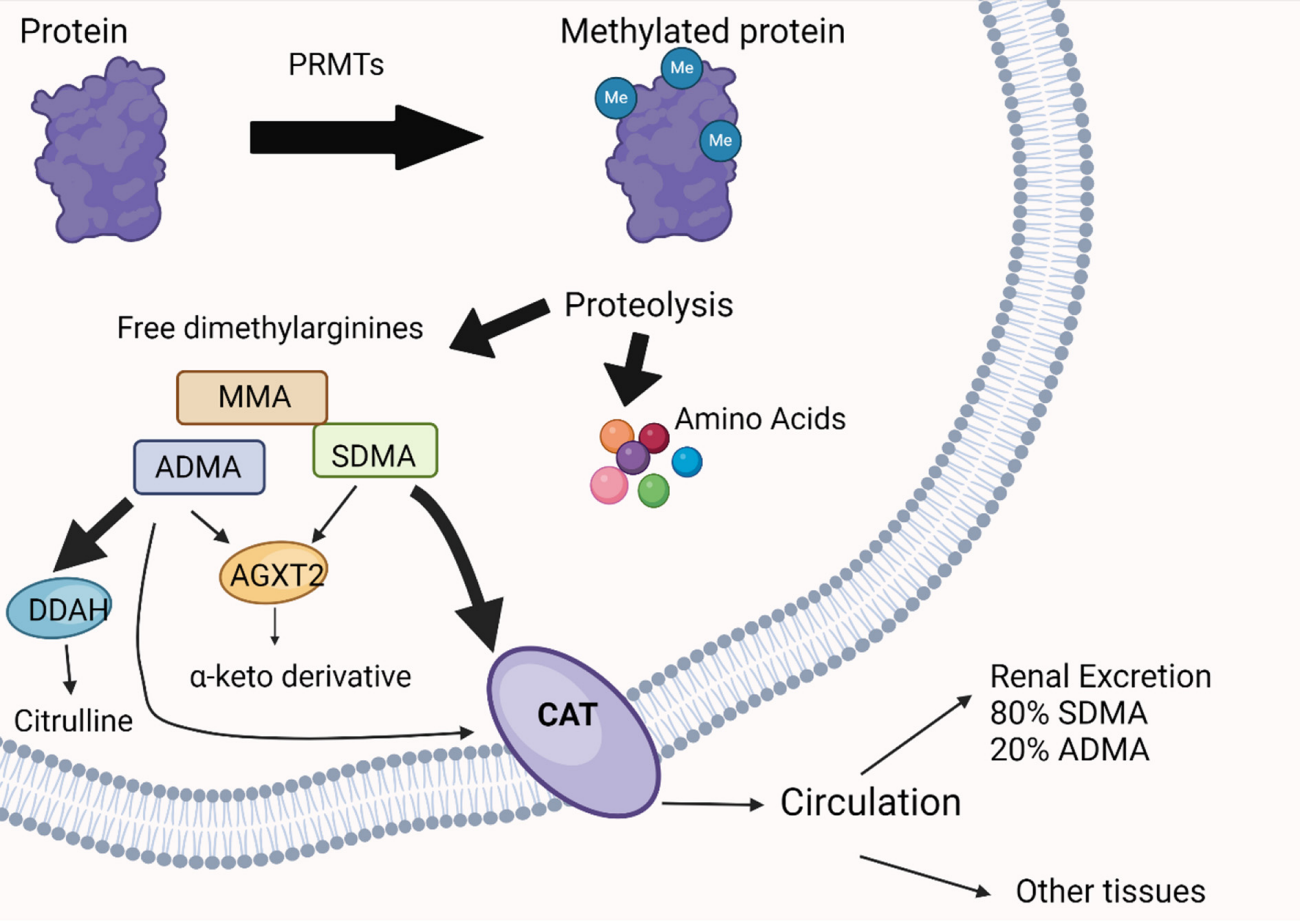
Synthesis, metabolism, and elimination of dimethylarginines Dimethylarginines are produced by the methylation of arginine residues during post translational modification of proteins by PRMT’s enzymes and are released by proteolysis. All types of PRMT (I-III) can produce L’NMMA, type I PRMTs produce ADMA and type II PRMTs produce SDMA. In the cytosol, DDAH1 and DDAH2 enzymes metabolise ADMA into citrulline but have no effect on SDMA. AGXT2 can convert both SDMA and ADMA into α-keto-derivatives. SDMA and ADMA can pass through the cell membrane through the CAT-2B transporter to access circulation, other organs and be eliminated via the kidneys. Created with BioRender.com.

Methylated analogues of L-arginine are produced in all cells by the post-translational modification of arginine residues in proteins by the protein-arginine methyltransferase (PRMTs) family of enzymes [32] (Figure 1). There are three types of PRMT, all of which are capable of monomethylation of arginine residues, resulting in the generation of NG-mono-methylated-L-arginine (L-NMMA). Type I and type II PRMTs additionally catalyse the addition of a second methyl group, leading to the formation of ADMA and SDMA, respectively [33]. Free L-NMMA, SDMA and ADMA are released upon protein degradation and can be transported into the circulation or taken up into other cells via cationic amino acid transporters, which are also responsible for L-arginine transport [34]. ADMA, SDMA and L-arginine are also transported across cell membranes via the sodium-coupled citrate transporter NaCT/Nact (SLC13A5) which is highly expressed in the liver and brain [35].

ADMA and L-NMMA are both competitive inhibitors of NOS and, therefore, have the potential to cause significant (patho)physiological effects [31]. ADMA is present in the blood at an approximately 5- to 10-fold higher concentration than L-NMMA and, thus, is generally viewed as the more biologically relevant of the two [31,36]. By contrast, SDMA, which is present in the circulation at a similar concentration to ADMA (∼0.4–0.6 μg/L) [37], is not considered a direct inhibitor of NOS [31]. ADMA levels are tightly regulated by the activity of dimethylarginine dimethylaminohydrolase enzymes (DDAH1 and DDAH2), which hydrolyse ADMA to citrulline and dimethylamine. Approximately 80% of ADMA is metabolized by the DDAH enzymes and the vast majority of the remaining 20% of ADMA is eliminated in the urine by the kidney [38]. SDMA, in contrast, is not a substrate of the DDAH enzymes and, is almost exclusively eliminated through urine (>90%) [39]. Alanine-glyoxylate aminotransferase 2 (AGXT2) – a mitochondrial aminotransferase predominantly expressed in the kidney, with some activity reported in the brain and liver – can convert both ADMA and SDMA into α-keto-derivatives, although the full significance of its role in methylarginine metabolism is still to be determined [40]. Nevertheless, the fact that plasma SDMA levels are significantly increased by disruption of the AGXT2 gene in mice and in humans are associated with single nucleotide polymorphisms (SNPs) in the AGXT2 gene suggests its contribution is not negligible [41,42].

## Proposed biological effects of SDMA

While SDMA itself does not directly inhibit NOS activity, a small number of *in vitro* studies suggest that SDMA may reduce the availability of L-arginine as a substrate for NOS, which may subsequently lead to NOS uncoupling and reduced NO generation. Early studies suggest that SDMA competes with L-arginine for cellular uptake via the cationic amino acid transporter-2B (CAT-2B) and drives out intracellular L-arginine [43,44], at least when present in the 1–10 mM concentration range. Treatment of human umbilical vein endothelial cells (HUVEC) with 2–100 µM SDMA led to dose dependent inhibition of NO production and increased reactive oxygen species (ROS), which could be reversed by L-arginine supplementation [32]. In another study, SDMA at a concentration of 0.1 µM did not affect resting NO generation in glomerular endothelial cells but impaired VEGF-induced eNOS activation at Ser1177, the Akt (protein kinase B) phosphorylation site, leading to increased superoxide production and decreased NO synthesis [45]. These effects could be reversed by supplementation of cells with excess L-arginine, suggesting they were caused by the inhibition of L-arginine uptake by SDMA [45]. Notably, as ADMA is structurally similar to SDMA, it is transported into and out of cells via similar mechanisms and will also compete with L-arginine for cellular uptake [43]. Therefore, changes in the abundance of SDMA might also influence ADMA uptake and thus its (patho)physiological effects (and vice versa). Indeed, SDMA (100 µM) impaired CAT-1 mediated uptake of L-arginine [46] and impaired the uptake of L-arginine, ADMA and L-homoarginine (L-hArg, an arginine derivative that can facilitate NO production by acting as a weak NOS substrate) by human and mouse NaCT/ Nact [35]. Given that ADMA competes with L-arginine for the active site of NOS, effects of SDMA on L-arginine availability may also influence the ADMA–NOS interaction. As such, the relative abundance of SDMA to other arginine derivatives may determine its biological impact rather than SDMA levels. In addition, it is also important to note that with the exception of severe kidney disease, where circulating levels of SDMA can reach 2–3 µM [47–51], relatively small differences in SDMA (0.1–0.2 µM) are associated with vascular risk factors and disease outcomes. In this context, it is questionable whether small changes in SDMA would affect the cellular uptake of arginine to the extent that NOS function is impaired when the concentration of L-arginine in plasma (41–114 µM) far exceeds that of SDMA [52,53]. Thus, it is conceivable that some of the biological effects of SDMA could be mediated by a yet undefined pathway and/or receptor, independent of NOS. In support, findings from a recent study suggests that SDMA can bind the amino acid binding pocket of the calcium sensing receptor (CaSR) and antagonise positive allosteric modulation by mediators such as phenylalanine and ADMA [54].

Evidence suggests that SDMA may also influence pro-inflammatory functions of immune cells, although the mechanisms involved are not well characterized. Micromolar (3–6 µM) SDMA acutely augments the oxidative burst activity of cultured human THP-1 monocytes via the activation of store-operated calcium channels [55], and promotes the generation of cytokines IL-6 and TNF-α, likely through the activation of NF-κB [49]. Moreover, treatment of monocytes and granulocytes with 6.1 µM SDMA up-regulates the expression of surface adhesion molecules involved in monocyte differentiation and endothelial adhesion (CD14 and alpha integrin components [CD11a, CD11b] on monocytes; CD18 on granulocytes) [56]. Interestingly, no changes in monocyte pro-inflammatory cytokines, NF-κB activation, or adhesion molecule expression (with the exception of CD14) were found with ADMA suggesting the pro-inflammatory effects of SDMA occur independent of NOS inhibitory mechanisms, and that SDMA may have (patho)biological actions distinct from those of ADMA [49,55]. In the same study, SDMA, but not ADMA, inhibited the proliferation of HUVEC *in vitro*, suggesting SDMA may also modulate endothelial repair mechanisms [56]. Future work using primary endothelial cells is needed, however, to substantiate the significance of these findings and to explore the potential mechanisms involved. Most recently, a study in a mouse model of renal insufficiency showed that intra-renal administration of 25 µmol/kg SDMA downregulated STAT4, a transcription factor with a key role in immune cell function including in T-cell polarization and cell-mediated immune responses [57].

**Figure 2:**
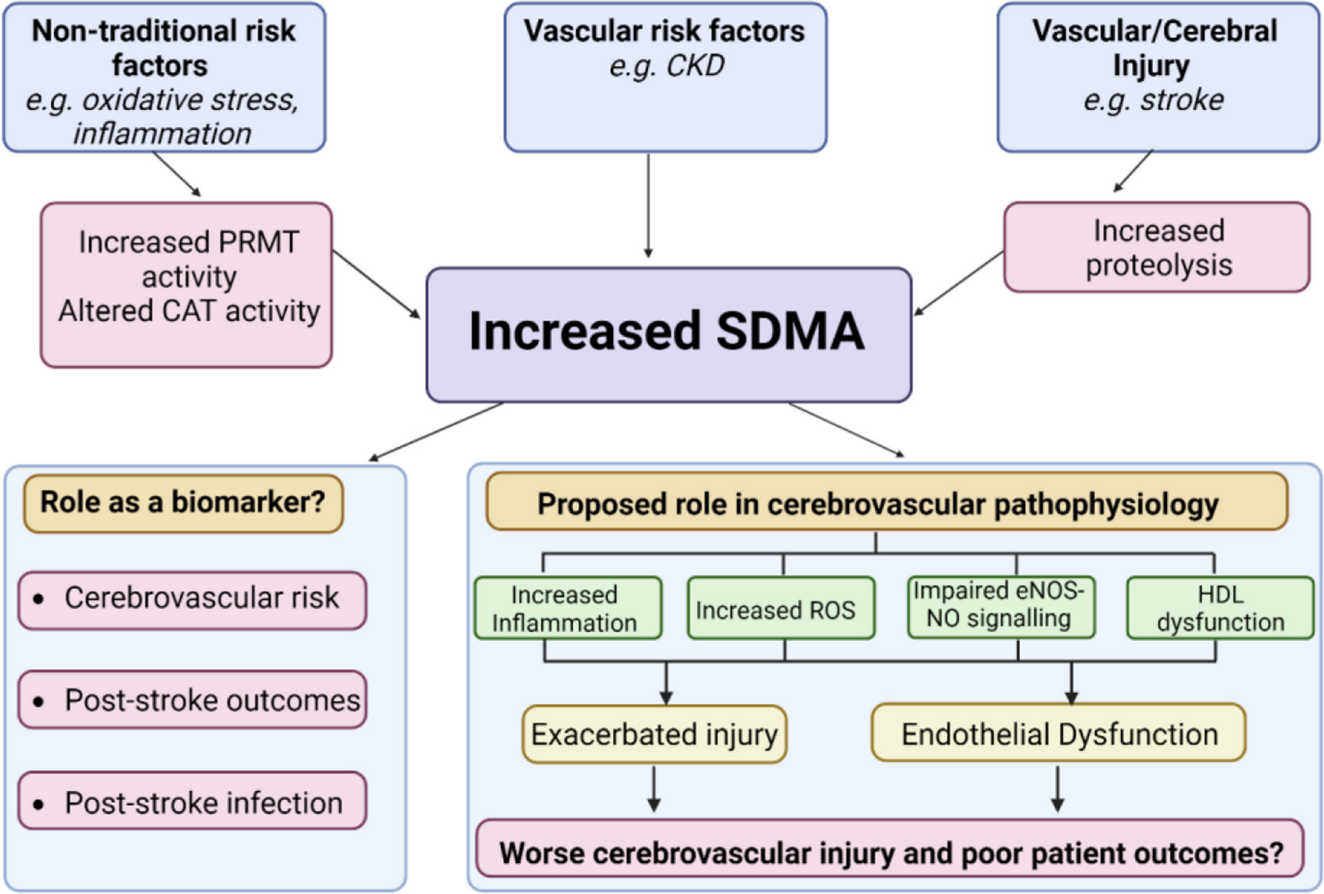
Proposed function of SDMA as a marker and mediator of cerebrovascular pathology Traditional and non-traditional cardiovascular risk factors and proteolysis secondary to vascular or cerebral injury may contribute to increased circulating levels of SDMA. Clinical evidence suggests that elevated circulating SDMA may indicate enhanced cerebrovascular risk and poor outcomes following ischaemic and haemorrhagic stroke based on a small number of studies [20–24,60,61,62]. However, emerging pre-clinical data additionally suggest SDMA itself has biological activity and could contribute to cerebrovascular injury and poor outcomes post injury via inhibitory effects on eNOS signalling [32,45], harmful effects on HDL function [63,64] and the exacerbation of inflammation and oxidative stress [51,57–59]. Created with BioRender.com.

The pathophysiological functions of SDMA may be mediated, at least in part, through its accumulation in high-density lipoprotein (HDL) particles, which occurs in CKD when circulating SDMA is markedly elevated [58]. *In vitro*, supplementation of HDL with SDMA activates Toll-Like receptor 2 (TLR-2) in human aortic endothelial cells (HAEC), which in turn activates the ROS generating Nox-NADPH oxidases leading to oxidative stress and decreased NO bioavailability [58,59]. SDMA-supplemented HDL decreased Akt phosphorylation, which is accompanied by increased eNOS phosphorylation at the inhibitory Thr495 site and decreased phosphorylation at the activating Ser1177 site, whilst SDMA alone (in contrast with earlier reports [32,45]) had no effect on NO production by HAEC [58]. Unlike HDL alone, SDMA-HDL inhibited endothelial cell migration and repair in an *in vitro* wound-healing assay and carotid artery injury model, respectively [58,59]. SDMA-HDL was unable to reduce TNF-α-induced endothelial VCAM-1 expression and enhanced monocyte adhesion in response to TNF-α [59]. Altogether, this suggests that accumulation of SDMA in HDL can disrupt the beneficial, anti-inflammatory effects of HDL and transform it into an abnormal lipoprotein capable of triggering inflammation, oxidative stress and endothelial cell dysfunction. Indeed, the accumulation of SDMA in HDL is proposed to be a marker and/or mediator of pre-mature cardiovascular disease in patients with CKD [59]. However, it remains unclear whether the effect of SDMA on HDL is specific to uraemic conditions or is also implicated in cardiovascular conditions without underlying renal dysfunction (Figure 2).

Although our overall understanding of SDMA still lags behind work on ADMA, these studies collectively provide evidence that SDMA is, in fact, biologically active and might have important (patho)physiological effects on peripheral vascular cells. However, there is a lack of studies showing (patho)physiological effects on cerebral vascular cells. Furthermore, the extent to which SDMA influences vascular function *in vivo* during health and disease is less clear, and there is a lack of experimental data exploring whether causal links exist between SDMA and vascular pathology. In addition, in several of the aforementioned studies, the concentration of SDMA used exceeds SDMA levels in the circulation in (patho)physiological conditions. Therefore, in order to appreciate the biological significance of these findings, further work is required to confirm that the reported effects of SDMA *in vitro* can occur with modest changes in SDMA concentration and that they are retained in the more complex *in vivo* setting.

## SDMA and (cerebro)vascular risk factors

### Chronic kidney disease and metabolic-related diseases

Several clinical studies describe an association of circulating levels of SDMA with (cerebro)vascular risk factors including CKD, atherosclerosis, hypertension, age and metabolic status. However, these associations are not consistently identified across studies, possibly reflecting the inherent complex interactions between the risk factors, which typically coincide together. Also, these risk factors share common pathological features of endothelial dysfunction, oxidative stress, inflammation and altered metabolic status, making it challenging to define the relationship of SDMA to any singular factor. What is clear, however, is that due to its dependence on kidney function for its elimination, SDMA shows a strong, inverse relationship with estimated glomerular filtration rate (eGFR) [53]. Although elevations in SDMA are associated with progression to end-stage renal disease [63], causal involvement of SDMA in kidney disease is not well defined. In healthy mice, SDMA does not directly affect renal function or promote the development of renal fibrosis [64], but it may contribute to endothelial dysfunction and increase systolic blood pressure via accumulation in HDL [58]. In support, high circulating SDMA levels predict endothelial dysfunction in the peripheral circulation of CKD patients, even after adjustment for renal function as measured by eGFR [65]. Paradoxically, a recent study demonstrated that direct intrarenal administration of SDMA to mice that underwent unilateral urethral obstruction or ischaemia–reperfusion injury attenuated renal tubulointerstitial fibrosis, whereas it had no effect on the kidneys of mice that received a sham procedure [57]. Whilst these findings conflict with the notion of SDMA as a uraemic toxin, they highlight that the biological function of SDMA may depend on the pathophysiological context.

Increases in circulating SDMA levels with advancing age are reported, although the extent to which this can be attributed to a simultaneous worsening of kidney function is not fully understood [21,65–67]. SDMA levels may also be increased in patients who have a greater BMI or waist circumference (index of abdominal adiposity) [68], although this has not been consistently reported in all studies [67,69,70]. Conflicting data are available on the relationship between circulating SDMA levels and insulin resistance and glycaemic control. In a population of non-diabetic subjects, SDMA was negatively correlated with insulin resistance in Caucasian, but not African, subjects adjusted for cofounders such as renal function, age, and BMI [71]; and in a cohort of atherosclerotic patients and age-matched controls, circulating SDMA levels were lower in insulin resistant than insulin sensitive individuals [72]. A third study reported lower SDMA levels in type 2 diabetic patients with poor glycaemic control and an inverse association of SDMA with HbA1c and fructosamine levels after application of eGFR-adjusted correlation coefficients [73]. In contrast, Atalar *et al*. reported greater circulating SDMA levels in patients with uncontrolled type 2 diabetes compared with non-diabetics and patients with controlled diabetes [74], and in a group of obese, but otherwise healthy adults, SDMA was positively correlated with insulin resistance and triglycerides and negatively correlated with HDL cholesterol [68]. However, neither study considered the potential confounding effect of renal function on SDMA levels. Together the findings suggest that SDMA may be influenced by metabolic status, but that the relationship is complex and requires further exploration. Certainly, it is reasonable to expect that changes in metabolic status might impact on the release of free SDMA levels in the circulation, for example through changes in the rate of protein catabolism. Moreover, reduced PRMT activity has been reported in type 2 diabetes melitus [75] and, *in vitro*, hyperinsulinaemia is reported to alter CAT-2B activity [76], which could affect SMDA levels by influencing SDMA generation and/or its transport between the circulation and cellular compartments.

### Hypertension

The relationship between SDMA and hypertension is also elusive, and only described in a small number of studies. Reports exist of elevated SDMA levels in children or adults with hypertension [77,78], though, these studies either did not account for differences in renal function between hypertensive and normotensive subjects or did not identify SDMA as an independent predictor of hypertension following adjustment for co-variables in multivariate analysis. Similarly, elevated circulating SDMA levels described in AF patients with a history of hypertension [79], and in hypertensive individuals with pre-eclampsia [80], rheumatoid arthritis [81], and sleep apnoea [69], were also not evaluated in the context of potential confounding factors such as renal function. Another recent study of hypertensive patients found no significant correlation of SDMA plasma levels with 24 h ambulatory blood pressures and pulse wave velocity [82], a measure of arterial stiffness and indicator of endothelial function [83]. Experimental evidence of a link between SDMA and arterial blood pressure is also lacking. Administration of SDMA to endothelium-intact, but not endothelium-denuded, mouse aortic rings, was found to have a pro-contractile effect, however this only became evident when the SDMA concentration was raised to 30 µM (10 times greater than the concentration of ADMA required to elicit a similar response) [77]. Moreover, although SDMA has been tentatively linked with vascular remodelling (albeit of the thoracic aorta) in spontaneously hypertensive rats [84], continuous delivery of pathophysiological levels of SDMA to mice has no effect on systolic blood pressure [64]. Although this could be affected by the use of young healthy mice, which clearly differs substantially from the complex pathological setting associated with hypertension development. However, in a recent study of spontaneously hypertensive rats, plasma SDMA levels were comparable between 16-week-old normotensive and hypertensive groups and, at 12 months were paradoxically reduced in the hypertensive group [85]. When taken together, these studies suggest that the reported association between SDMA and hypertension may be explained by the presence of shared risk factors rather than a direct effect of SDMA on hypertension development or blood pressure regulation. However, this conclusion does remain tentative as it is based on a small number of clinical and experimental studies, many of which do not primarily focus on dissecting the specific contribution of SDMA to hypertension.

### Atherosclerosis

Several studies have evaluated the relationship between circulating SDMA and carotid atherosclerotic pathology with mixed findings reported. In asymptomatic subjects with normal renal function, high circulating SDMA levels positively correlated with carotid intima-media thickness (cIMT, a marker of atherosclerotic development) and the presence of carotid atherosclerotic plaques [86]. Similarly, analyses of adults from the Study of Health in Pomerania cohort identified a positive association between SDMA and cIMT in multivariable analysis, which remained significant after adjustment for renal function, age, sex, and cardiovascular risk [87]. In addition, in patients with a history of ischaemic stroke, high SDMA or a low ratio of L-hArg to SDMA in the circulation were both identified as significant, independent predictors of internal carotid artery (ICA) stenosis but not cIMT on regression analysis [88]. Similarly, in a recent study of patients with a history of ischaemic stroke and >50% carotid stenosis alongside normal control subjects, metabolomic profiling coupled with machine learning demonstrated a benefit of including SDMA in predictive models aimed at detecting carotid artery stenosis [89]. This echoes findings in non-carotid locations (e.g., coronary arteries and aorta) where elevated circulating SDMA levels were independently associated with atherosclerotic lesion size [32,90]. Another study reported a significant independent association between percentage change in cIMT and SDMA levels over a 3-year study period in black but not white men, in multivariate analysis [91]. However, in contrast, a recent study of black South African subjects did not identify a significant association between baseline circulating SDMA levels and cIMT at the end of the 5-year study period in either univariate or multivariate analysis [92]. A number of other studies have reported a positive correlation between circulating SDMA and carotid atherosclerotic lesions or ICA stenosis in specific disease cohorts including in women with pre-eclampsia [80], patients with a history of ischaemic stroke [93] and patients with type 2 diabetes mellitus [94,95]. However, they either did not evaluate the potential confounding effects of renal function and vascular risk factors [80,93,94] or found that the significance of the association was lost once covariates were included in the analyses [95]. The collected evidence so far points towards a potential link between SDMA and carotid atherosclerotic pathology, but the extent to which this might depend on the existence of vascular risk factors and renal function is unclear. Given atherosclerosis is a major risk factor for stroke, and is linked to vascular and neurodegenerative causes of cognitive impairment and dementia [96,97], untangling the relationship between SDMA and atherosclerosis, and how it influences cerebrovascular risk may be important for the future diagnosis and management of atherosclerotic-induced cerebrovascular disorders.

### Atrial fibrillation

Conflicting data exists on the relationship between SDMA and atrial cardiomyopathy, another predisposing factor for thromboembolism and ischaemic stroke. Analysis of the Gutenberg Health Study revealed independent associations of SDMA levels with atrial remodelling and alterations in electrical conductance in a population-based cohort [36]. Moreover, in a study of individuals with ischaemic stroke, plasma SDMA positively correlated with left-atrial volume index, independent of thromboembolic risk and eGFR suggesting links between SDMA and atrial pathology [98]. Other authors report links between circulating SDMA and AF, for example, Büttner et al. reported that SDMA levels were generally higher in individuals with AF at baseline than those in sinus rhythm (SR) in an AF cohort, and noted that restoration of SR was associated with a marked decrease in SDMA levels at follow-up with no change in eGFR [99]. The authors of this study hypothesized that SDMA may directly contribute to AF pathogenesis through its HDL modulatory properties []. Indeed, HDL function is markedly altered in AF, and SR restoration improves HDL [100] and endothelial function [101]. However, several studies (including an analysis of the Framingham Offspring cohort [102] and a study of patients with ischaemic stroke [103]), have found that SDMA is not significantly altered in incident or prevalent AF once vascular risk factors or thromboembolic risk had been accounted for [36,98]. Similarly, a study of three independent cohorts of ischaemic stroke patients of various stroke aetiologies (e.g., large-artery and small-vessel atherothrombotic; cardioembolic), reported a lower circulating L-hArg/SDMA ratio in subjects with AF compared with those without AF, that was no longer significant after adjustment for age, sex, and vascular risk factors [88]. Thus, whilst some studies suggest that increased SDMA may occur alongside AF or atrial remodelling, it seems likely that SDMA may be a mere bystander correlated with shared cardiovascular risk factors rather than a direct mediator of atrial pathology. Nevertheless, the mechanisms that drive AF pathogenesis such as endothelial dysfunction, renal insufficiency and subclinical atherosclerosis and how they relate to SDMA is not well understood nor fully captured by associative data alone. Further research is clearly necessary to define the relationship between SDMA and atrial cardiomyopathy and explore whether a causal interaction exists.

## Prediction of cerebrovascular events

Considering the number of studies that report elevated circulating levels of SDMA with a range of cerebrovascular risk factors, it is perhaps not surprising, higher levels of SDMA coincide with increased risk of cardiovascular events, including stroke, across different patient populations. A study of non-dialysis CKD individuals identified in multivariate analyses that patients with circulating SDMA levels in the highest tertile were at increased risk of an atherosclerotic cardiovascular event (including stroke) during the ∼5-year follow-up period, after adjustment for renal function, age, sex, diabetes mellitus, smoking history, total cholesterol and existing cardiovascular disease [63]. In a recent case–control study, plasma SDMA levels were higher in patients with acute myocardial infarction and were a significant, independent predictor of a major adverse cardiac event (MACE; composite of cardiovascular death, non-fatal MI and non-fatal stroke) during the median 3.5 year follow-up period [104]. Hov et al. similarly reported a positive association between circulating SDMA levels and acute cardiovascular events (which included myocardial infarction, ischaemic stroke, and transient ischemic attack [TIA]) in patients with moderate CKD, respectively, in univariate analysis, however, this was not significant on multivariate analysis [105]. In two independent evaluations of type 2 diabetic patients, elevated SDMA levels predicted risk of cardiovascular events (including stroke) at follow up, but in both cases, significance was also lost after multivariate analysis [106,107]. In a longitudinal study of patients with type 2 diabetes mellitus and microalbuminuria, high baseline SDMA levels was an independent predictor of incident cardiovascular disease (including stroke), all-cause mortality, and decline in eGFR of >30% (independent of baseline eGFR) during the 6-year study [108]. However, when SDMA was added to a predictive model alongside traditional cardiovascular risk factors, it did not enhance risk prediction. Most recently, however, in a study of patients with diabetes mellitus and mild-to-moderate CKD, plasma SDMA showed no significant association with incident atherosclerotic cardiovascular disease (time to MI, stroke or peripheral artery disease event) [109]. Together these studies suggest that elevated SDMA levels are indicative of an increased risk of cardiovascular events including stroke; however, whether this relationship is independent of renal function, or the presence of other vascular risk factors is less clear. Also, given these studies frequently evaluated combined cardiovascular outcomes, further studies are needed to investigate whether SDMA levels are associated with incident cerebrovascular events in isolation from other cardiovascular events. A meta-analysis of existing data would also enhance our understanding of how circulating SDMA relates to stroke risk.

Within cohorts of patients at risk of stroke, for example, those with AF or a history of ischaemic stroke, circulating SDMA levels may help stratify the risk of a thromboembolic event or stroke recurrence, respectively. In ischaemic stroke patients, Cordts et al. reported an association of a low circulating L-hArg/SDMA ratio with a greater stroke risk according to the CHA_2_DS_2_-VASc score [88]. SDMA levels were also positively correlated with increased risk prediction scores (CHA_2_DS_2_-VASc and Essen stroke risk score [ESRS]) in AF and ESUS (embolic stroke of undetermined source) patients, and there was an inverse association of these scores with the L-Arg/SDMA ratio [103]. In a sub-study of the ARISTOTLE (Apixaban for Reduction in Stroke and Other Thromboembolic Events in Atrial Fibrillation) trial in AF patients, SDMA levels correlated with CHADS_2_ and CHA_2_DS_2_-VASc scores of stroke risk [79]. Although SDMA levels were not independently associated with stroke risk and systemic embolism, they were associated with the risk of major haemorrhage and death in anti-coagulated patients after adjustments for clinical risk factors (e.g., age, sex, hypertension, diabetes, and creatinine clearance).

Interestingly, in patients with acute ischaemic stroke, elevations in circulating SDMA may be associated with specific stroke aetiologies, particularly cardioembolic stroke. Wanby et al. reported an independent association of SDMA levels with cardioembolic infarction but not non-cardio-embolic infarction in patients with acute cerebrovascular disease [93]. A lower L-hArg/SDMA ratio was also described in patients with stroke due to large vessel disease or cardioembolism compared to stroke related to cerebral small vessel disease [88]. In contrast, Schulze et al. found no difference in SDMA levels with stroke subtypes classified according to the TOAST system [21]. SDMA levels also did not differ between cardioembolic and non-cardioembolic stroke types in a study by Brouns et al. but this may reflect the fact that SDMA levels were measured in CSF rather than in the circulation, and thus may be less influenced by cardioembolic pathology [22]. The relationship between SDMA and stroke aetiology clearly requires further clarification, nevertheless, the fact that SDMA levels have been linked to thromboembolic risk scores, atrial pathology and cardioembolic stroke across different studies creates a growing picture of pathological landscape that SDMA may be particularly implicated in.

## SDMA and ischaemic and haemorrhagic stroke

Several studies comprising of relatively small cohorts of ischaemic stroke patients have shown that concentrations of SDMA in the plasma or CSF are elevated during the acute phase after stroke onset [22,23,110]. Plasma SDMA levels are reported to increase as early as 6 h after ischaemic stroke and remain elevated for at least the first three days after stroke onset [23,110]. By contrast, Bladowski et al. observed that plasma SDMA levels in the 7 days post-stroke did not significantly differ from levels in healthy control patients; however, the authors suggested this might be explained by the inclusion of patients with milder strokes in the study population compared with other studies [111]. Notably, available clinical data support a positive association of post-stroke SDMA levels with poor short- and long-term patient outcomes, although the data are limited by the small sample sizes used, and often the relatively low absolute number of primary events recorded within the study timeframe [20,22,23,110]. In acute ischaemic stroke patients, elevated circulating SDMA, but not ADMA, was shown to be a marker for adverse cardiovascular events or death in the 30 day follow-up period, a correlation that was linked to the relationship between SDMA and renal function [20]. However, the statistical power of this study was low due to small sample size of 137 patients, of which only 25 experienced the primary composite endpoint. In addition, the cohort comprised of patients who suffered relatively mild stroke, which may affect its external validity. In a small number of ischaemic stroke patients, increased circulating SDMA levels prior to thrombolysis correlated with an unfavourable outcome at 90 days post-stroke in univariate analysis, however, was not an independent determinant of outcome once covariates such as age and diabetic status were accounted for [60]. In contrast, in a cohort of 67 patients, Worthmann et al. reported a significant positive association of SDMA or ADMA levels in the first 3 days post-ischaemic stroke with a poor outcome (modified Rankin scale [mRS]) after the 90 day follow up period even after correction for eGFR, stroke severity on admission (National Institutes of Health Stroke Scale [NIHSS]), and hypertension [23]. Schulze et al. showed that elevated circulating SDMA levels, but not ADMA, predicted total mortality in the 7 years after acute stroke in multivariable analysis in a cohort of 394 acute stroke patients, suggesting that assessment of baseline SDMA levels may forecast outcomes over a prolonged period (years rather than months) [21]. A decreased baseline L-hArg/SDMA ratio was also shown to independently predict long-term all-cause mortality, in addition to short-term outcomes (adverse events, neurological impairment, and disability) in a study of two prospective cohorts of acute stroke patients [112]. CSF levels of SDMA post-stroke may also predict stroke outcomes, as a study by Brouns et al. reported that CSF SDMA and ADMA levels in the first 24 h after stroke onset positively correlated with stroke severity (National Institutes of Health Stroke Scale [NIHSS]) and poor functional outcomes (need for institutionalization at discharge and a greater degree of dependency at 90 days post-stroke). However, in multivariable analysis, patient characteristics (e.g., age) and vascular risk factors were superior predictors of outcomes than CSF dimethylarginines [22].

Similar to ischaemic stroke, there is some evidence that SDMA levels may also be relevant in the setting of haemorrhagic stroke, which may warrant further exploration. A recent study of patients with intracerebral haemorrhage (ICH) showed that intrathecal, but not plasma SDMA, is increased in the 10 days following haemorrhage [113]. In a small study, serum SDMA levels were comparable between 20 acute ICH and 30 control patients. However, SDMA levels were elevated in ICH patients with larger haematomas and peri-haematomal oedema volumes compared with those with smaller bleeds and oedema volumes, and this was associated with poorer neurological outcomes at 90 days [24], although the statistical power of this study is low due to small sample sizes. SDMA levels in the CSF were also found to be increased during the first 10 days following subarachnoid haemorrhage (SAH) compared with control subjects [114]. Furthermore, in a separate study comprising of 34 SAH patients, elevated CSF SDMA levels correlated with poorer neurological status on admission, as well as worse neurological outcomes 3 months later, independent of vascular risk factors [115]. Moreover, patients that developed delayed cerebral ischemia (DCI) had higher levels of CSF SDMA in the first 6 days following hospitalization, than non-DCI patients [115]. However, the predictive power of SDMA for DCI was lost in the final adjusted model, which included adjusting for clinical severity and traditional risk factors including a history of hypertension, age and sex. Similar findings were recently reported in a retrospective study of patients with severe SAH, which used a combination of CSF metabolite profiling and machine learning to identify metabolites that were associated with aneurysmal SAH outcomes [116]. Higher SDMA CSF levels were significantly associated with poor neurological outcomes at discharge and at the end of the 90 day follow-up period but were not predictive of radiological evidence of vasospasm. Taken together, the studies suggest that CSF SDMA may predict neurological outcomes following SAH but may not be linked to the development of vasospasm and DCI. However, as both these studies comprised a small number of patients with severe SAH, further work is needed to corroborate these findings and confirm their generalisability to the wider population of SAH patients.

## Limitations of current evidence

Clinical data have highlighted potential associations between SDMA and cerebrovascular disease, however, our understanding of why these associations exist is limited by low study sample sizes, inconsistent adjustment for confounders and a lack of interventional studies (particularly within a pre-clinical context) to provide direct causal evidence for a role of SDMA. Considering the close correlation of plasma SDMA with renal function [61], it is conceivable that SDMA is solely a marker of decreased renal capacity. As such, the reported associations of SDMA with atherosclerosis, atrial fibrillation and hypertension [80,86,94,99] might be largely explained by the fact that co-morbid renal disease is often present in these populations [62,117]. Likewise, CKD is a powerful risk factor for cerebrovascular disease, and CKD or acute kidney injury are prevalent in patients post-stroke [118], and are predictors of poor outcomes [119]. SDMA may be more sensitive to changes in kidney function compared with existing markers [120] and so it is possible that the apparent independent associations of SDMA with cerebrovascular disease are influenced by subclinical renal dysfunction not accounted for by GFR estimates in regression analyses [121]. Indeed, many of the aforementioned studies, evaluate GFR based on serum creatinine-based formulae, which can be insensitive to minor changes in renal function, and can vary in accuracy depending on the algorithm used [122]. Future studies that use plasma or urinary clearance of an exogenous filtration marker to measure kidney function or use combined kidney biomarkers may be a superior means to evaluate the confounding effects of renal function, but may not be feasible for many studies and may still be influenced by non-kidney determinants [122]. Use of control groups that are matched for renal function and exclusion of patients with abnormal renal function may also help minimise confounding effects.

Beyond renal function, the factors regulating circulating levels of SDMA are ill-defined. However, findings from genome-wide association analysis linking SDMA to variants in the *AGXT2* gene, suggests that SDMA metabolism plays a role and is, at least partly, a heritable trait [42]. Mendelian randomization could also be employed in future studies to reduce confounding effects due to shared risk factors and potentially provide genetic evidence of any causal relationships that exist [123]. However, such analyses are complicated by the fact that AGXT2 has several substrates aside from SDMA [124]. Future work using pre-clinical stroke models will be pivotal to address whether a causal interaction of SDMA with brain injury and outcomes exists. Such studies will enable testing of the effects of manipulating SDMA levels (e.g. administration of exogenous SDMA) on stroke outcomes and facilitate dissection of any underlying mechanisms independent of potential confounding comorbidities.

However, regardless of whether SDMA plays a causal role in cerebrovascular disease, reports linking SDMA to poor stroke outcomes suggest it could serve as a valuable prognostic biomarker. In this instance, the key question is not whether shared variables and confounding factors explain the relationship between SDMA and stroke, but rather whether measuring SDMA levels provide better outcome predictions than existing predictive models. This cannot be answered from the currently available studies and requires large, prospective studies with a prolonged follow up period to test the sensitivity and specificity of SDMA levels in predicting outcomes, and its utility in comparison with existing methods. In future, careful consideration should also be given to the method applied to measure SDMA. While most studies use mass spectrometry (MS) approaches such as liquid chromatography tandem mass spectrometry (LC-MS/MS) to measure methylarginines, separation of SDMA from ADMA can be challenging, and studies can vary significantly in concentrations of methylarginines reported [125]. Older LC-MS/MS measurement methods could be improved in terms of accuracy, reproducibility, and precision using separation methods based on specific fragmentation patterns rather than liquid chromatography [125]. Therefore, future studies of SDMA in cerebrovascular disease should employ robust LC-MS/MS methods that can be validated according to Clinical and Laboratory Standards Institute Protocols [125].

## Perspectives

Identification of SDMA as a predictor of stroke outcomes independent of eGFR and traditional vascular risk factors may provide the first tentative insight that SDMA may be more than a mere bystander and may have a biological role in (cerebro)vascular injury. Unfortunately, direct evidence of pathological effects in the cerebral circulation is lacking, as of the limited number of pre-clinical studies exploring pathophysiological functions of SDMA, none address its effects within the brain and its vasculature or examine its influence on stroke pathogenesis. Nevertheless, the potential of SDMA to promote inflammatory activation, oxidative stress, NO deficiency, and HDL dysfunction [32,49,55,56], indicated by the small number of *in vitro* studies available, suggests that elevated SDMA could incite or exacerbate endothelial dysfunction. This could be particularly important in settings of chronically elevated SDMA, such as CKD, where SDMA could enhance risk of stroke or other cerebrovascular complications by directly promoting cerebral vascular dysfunction or atherogenesis. Endothelial effects of SDMA could also contribute to the risk of thromboembolism by attenuating the normal inhibitory effects of eNOS-derived NO on platelet activation, aggregation and adhesion to the endothelium [126], potentially explaining data associating L-hArg/SDMA ratio with large vessel and cardioembolic stroke subtypes [88,93]. Recently, an eNOS expressing subtype of platelets, accounting for ∼20% of the total platelet population) was identified and proposed to negatively regulate thrombus formation [127]. Direct effects of SDMA on platelet function or platelet-derived NO have not been investigated; however, SDMA can accumulate in platelets and is associated with increased platelet aggregation capacity [128]. New data also indicate that intra-platelet SDMA is greater in patients who have experienced a recent acute ischaemic stroke compared with healthy control subjects [111], which could indicate the presence of a pro-thrombotic platelet state prior to stroke. Yet, while Schulze et al. reported a positive association between plasma SDMA and markers of platelet function (β-thromboglobulin) [21] other work paradoxically has suggested that plasma SDMA is inversely related to platelet aggregation capacity [104,129], signifying the existence of a complex relationship between SDMA, platelet function and stroke risk that requires further study.

As discussed in this review, limited clinical evidence suggests that SDMA levels are elevated in ischaemic and haemorrhagic stroke and may be associated with poor outcomes. However, our current understanding of peripheral-to-brain (and vice versa) SDMA dynamics is lacking and it is unclear if the reported elevations in SDMA concentrations within plasma (or CSF) stem from local proteolysis following cerebral injury, and/or whether this change reflects increased SDMA generation in the periphery. The rate of symmetric arginine methylation of cellular proteins and their subsequent proteolysis will also dictate the amount of free SDMA, at least intracellularly, with membrane transport of SDMA between cellular and extracellular compartments also likely to be important. Oxidative stress and inflammatory stimuli could contribute to elevated SDMA levels post-stroke by modulating the expression of PRMT enzymes and CAT transporters [130–132]. Indeed, ischaemic stroke can trigger a systemic catabolic (which includes muscle proteolysis) and pro-inflammatory profile, which could also explain, at least in part, elevated circulating SDMA levels [133].

Very little is known about SDMA levels in the brain, but a recent study of young healthy mice found approximately twice as much SDMA than ADMA in the prefrontal cortex and hippocampus, accounting for approximately 60% and 50% of total arginine derivatives measured in these areas, respectively [134]. SDMA was also reported to be higher than ADMA in bovine brain; however, in rat brain tissue, concentrations of the two dimethylarginines were comparable [135].The ability of SDMA to cross the BBB has also not been studied but it is reasonable to suspect that it may access the same BBB transcellular transporters used by L-arginine, for example via cationic amino acid transporters [136]. Thus, local elevations of SDMA, for example consequent to cerebral injury, may impact on circulating SDMA concentrations and vice versa. BBB disruption following ischemia might also facilitate paracellular transport [137] of SDMA between the periphery and the brain. Future work should assess whether SDMA levels also increase in rodent stroke models. This would validate cross-species relevance, while allowing direct measurement of SDMA in brain tissue to provide insights into its spatio-temporal dynamics and sources post-stroke.

Importantly, SDMA may correlate with poor stroke outcomes because it has a direct role in the exacerbation of cerebral injury. Of note, SDMA levels correlate with pro-inflammatory stimuli in the acute phase after ischaemic stroke [138], supporting a potential link between SDMA and post-stroke inflammation. The effect of SDMA on immune cell populations in the brain post-stroke has not been studied. However, the effects of SDMA on cultured human monocytes suggest it could increase ROS generation and the release of pro-inflammatory cytokines such as TNF-α and IL-6 [49,55]. This could trigger activation of microglia and astrocytes, driving their differentiation into pro-inflammatory M1 and A1 subtypes, respectively, which may worsen inflammation, increase neuronal damage, and impair repair processes [139]. Additionally, SDMA may up-regulate adhesion molecules on endothelial cells and circulating immune cells (as reported in cultured HUVECs and monocytes, respectively [56]), potentially increasing immune cell infiltration into the brain. Although the impact of elevated SDMA on BBB function has not been explored, a pro-oxidative, pro-inflammatory environment could disrupt tight junctions and increase BBB permeability, further promoting immune cell infiltration and tissue injury, and worse stroke outcomes [140].

The specific effects of SDMA on cerebral vessel function remain unstudied, but evidence from other cell types suggests it may reduce NO bioavailability and inhibit endothelial proliferation [32,45,56], which could contribute to microvascular dysfunction. After stroke, this might lead to impaired cerebral blood flow and reduced angiogenesis, resulting in increased ischemic injury and hindering the brain’s capacity for repair [18,141]. Future experiments assessing the impact of SDMA on the viability and proliferation of brain microvascular endothelial cells under oxygen and glucose deprivation (OGD) conditions would be a first step to provide insights into this area. Although associative clinical data align with a potential harmful role of SDMA, it is important to acknowledge that NO can have both beneficial and harmful effects in cerebral injury. Indeed, iNOS and nNOS aggravate injury, whereas eNOS is regarded as protective [142,143]. Therefore, through its NO limiting properties, SDMA might confer protection by limiting NO production from iNOS and nNOS.

Interestingly, SDMA has tentatively been associated with post-stroke infections, a frequent and often serious complication linked to morbidity and mortality [144]. In a relatively small cohort of patients, Molnar et al. found that circulating SDMA levels at 72 h following stroke onset were positively associated with post-stroke infections and circulating inflammatory markers, including the acute phase protein C-reactive protein (CRP) and the chemokine monocyte chemoattractant protein 1 (MCP-1) [110,145]. Recent work shows that infarct volume (or stroke severity) influences post-stroke infections [146]. Therefore, the association between SDMA and post-stroke infections in this cohort of patients may reflect a potential causal interaction with brain injury. Indeed, Molnar et al. showed that SDMA was positively correlated with S100 B levels, a biomarker of infarct size [110]. Nevertheless, it is conceivable that SDMA directly contributes to systemic immunosuppression after stroke [147] through its proposed effects on inflammatory cytokine expression and monocyte function [49,55,56]. Accumulation of SDMA in HDL particles could also contribute through the formation of dysfunctional, noxious HDL [58], which perhaps disrupts the innate anti-endotoxin function of HDL [148], resulting in a greater risk of infection post-stroke. Data connecting SDMA with post-stroke infections is clearly preliminary in nature, coming from studies of low-statistical power, and the mechanistic links are speculative. Future work is clearly needed to firstly substantiate the association in larger, longitudinal studies and secondly to test for a causal link.

Recent studies have begun to explore the relationship between SDMA and cognitive impairment. Reports exist of positive associations of circulating SDMA with cognitive impairment [149,150] and the presence of white matter lesions [66], which appear to be linked to patients’ age. In a cross-sectional study of Alzheimer’s disease, mixed-type dementia and vascular dementia patients, serum SDMA levels were not significantly different from subjects without dementia, once age was accounted for. However, SDMA levels were positively correlated with clinical dementia rating in all dementia patients and were inversely associated with mini-mental state examination (MMSE) in the vascular dementia subgroup, suggesting elevated SDMA levels may be linked to greater cognitive loss [149]. Although, after adjustment for age sex and BMI, SDMA remained an independent predictor of the clinical dementia rating only [149]. An inverse relationship between circulating SDMA and cognitive performance was also described in a small study of older adults without known cerebrovascular disease, but the significance of this association was lost following correction for co-variates including sex, BMI, and carriage of the APOE ε4 allele (the main genetic risk factor for Alzheimer’s disease) [151]. Interestingly, in the same study, SDMA levels were independently related to plasma levels of neurofilament light chain (NF-L) [151], an axonal cytoskeletal protein that acts as a biomarker for neurodegeneration and has previously been shown to relate to Alzheimer’s disease pathology and cognitive dysfunction [152]. Cerebrovascular abnormalities and cerebral injury are linked to both vascular and neurodegenerative causes of cognitive impairment and dementias. Therefore, whilst tentative at present, these associations provide a rationale to further explore the consequences of elevated SDMA levels for cerebrovascular and cognitive health.

A key challenge in the future of SDMA research will be devising means to manipulate SDMA levels to not only provide crucial causal evidence of SDMA's involvement in cerebrovascular health and/or disease but to also facilitate detailed explorations of the biological mechanisms involved. Furthermore, strategies to lower SDMA levels will be necessary to evaluate the translational impact of targeting SDMA, yet they are currently lacking. Reports that functional *AGXT2* variants affect SDMA levels suggests that targeting AGXT2 may be a viable option [42]. However, further research is needed to understand the regulation of AGXT2 activity, its impact on brain SDMA levels, and how AGXT2 expression or activity is modulate by disease states. Moreover, AGXT2 has several substrates aside from SDMA including ADMA, homoarginine and β-alanine [124]. As such, the therapeutic potential of targeting AGXT2 is currently unclear. A second approach to lowering SDMA levels could be to target symmetric protein arginine dimethylation by PRMTs, although this will affect protein methylation which may lead to off-target effects. Several type II PRMT inhibitors have been developed and have been tested in pre-clinical and clinical studies to treat various forms of cancer [153], but their therapeutic use in the context of cerebrovascular disease remains to be tested.

## Conclusion

Historically, SDMA has been considered an inert metabolite, of little biological relevance other than as a marker for impaired renal function. However, the emergence of pre-clinical data in recent decades demonstrating that SDMA has the capacity to affect NO bioavailability, promote oxidative stress and inflammation and precipitate HDL dysfunction, suggests that this view is out-dated, and calls for further research on the contribution of SDMA to pathophysiological processes. A causal role of SDMA in cerebrovascular disease has not been studied, but reports of associations between elevated SDMA levels and cerebrovascular risk factors and poor patient outcomes following ischaemic and haemorrhagic stroke, beyond renal function and traditional vascular risk factors, provides a strong justification for this work to take place. Pre-clinical studies detailing the effect of SDMA on cerebral vascular function, alongside studies evaluating the effect of altered levels of SDMA on stroke outcomes in experimental models with or without renal dysfunction will be crucial for understanding whether SDMA has a causal role in stroke pathogenesis and would be complemented by well-powered, longitudinal clinical studies in diverse stroke patients which explore the effect of potential confounders on SDMA levels and the utility of SDMA as a prognostic marker. A thorough dissection of the factors that influence cerebral and circulating of SDMA levels aside from renal function will also be key to our understanding of SDMA in this context and may offer additional insight into how SDMA levels may be manipulated for experimental or therapeutic purposes. A complex relationship clearly exists between SDMA and a number of stroke risk factors – including CKD, AF, atherosclerosis – that is challenging to reconcile based on currently available associative data alone. More research is clearly needed to address the contributions of SDMA in these conditions, and vice versa, and also should consider how these interactions feed into cerebrovascular risk.

## Data Availability

The submitted article is a review and does not have any associated data files. Therefore data sharing is not applicable in this context.

## Abbreviations

ADMA: asymmetric dimethylarginine
AF: atrial fibrillation
AGXT2: alanine-glyoxylate aminotransferase 2
BBB: blood–brain barrier
BMI: body mass index
CAT-2B: cationic amino acid transporter 2,
CBF: cerebral blood flow
cIMT: carotid intima-media thickness
CKD: chronic kidney disease
CRP: C-reactive protein
CSF: cerebral spinal fluid
DCI: delayed cerebral ischaemia
DDAH1: dimethylarginine dimethylaminohydroxylase 1
DDAH2: dimethylarginine dimethylaminohydroxylase 2
eGFR: estimated glomerular filtration rate
eNOS: endothelial nitric oxide synthase
ESUS: embolic stroke of undetermined source
FMD: flow-mediated dilation
GFR: glomerular filtration rate
HAEC: human aortic endothelial cell
HDL: high-density lipoprotein
HUVEC: human umbilical vein endothelial cell
ICH: intracerebral haemorrhage
iNOS: inducible nitric oxide synthase
L-hArg: L-homoarginine
L-NMMA: NG-mono-methylated-L-arginine
MI: myocardial infarction
MMSE: mini-mental state examination
mRS: modified Rankin Scale
NACT/nact: sodium-coupled citrate transporter
NO: nitric oxide
NOS: nitric oxide synthase
PRMT: protein arginine methyltransferase
SAH: subarachnoid haemorrhage
SDMA: symmetric dimethylarginine
SR: sinus rhythm
TIA: transient ischaemic attack
TLR-2: Toll-Like receptor 2
TNF-α: tumour necrosis factor alpha
VEGF: vascular endothelial growth factor
